# Design, Synthesis, Biological Activity and Molecular Dynamics Studies of Specific Protein Tyrosine Phosphatase 1B Inhibitors over SHP-2

**DOI:** 10.3390/ijms140612661

**Published:** 2013-06-17

**Authors:** Su-Xia Sun, Xiao-Bo Li, Wen-Bo Liu, Ying Ma, Run-Ling Wang, Xian-Chao Cheng, Shu-Qing Wang, Wei Liu

**Affiliations:** 1 Tianjin Key Laboratory on Technologies Enabling Development of Clinical Therapeutics and Diagnostics (Theranostics), School of Pharmacy, Tianjin Medical University, No.22 of Qixiangtai Road, Heping, Tianjin 300070, China; E-Mails: yuhuashixbd@163.com (S.-X.S.); acton.lee@gmail.com (X.-B.L.); bowenliu2008@163.com (W.-B.L.); maying@tijmu.edu.cn (Y.M.); chengxianchao@tijmu.edu.cn (C.-X.C.); wangshuqing@tijmu.edu.cn (S.-Q.W.); 2 Tianjin Institute of Pharmaceutical Research (TIPR), Tianjin 300193, China; E-Mail: liuweitianjin2010@163.com

**Keywords:** PTP1B, SHP-2, synthesis, imidazolidine, activity, molecular dynamics

## Abstract

Over expressing in *PTPN1* (encoding Protein tyrosine phosphatase 1B, PTP1B), a protein tyrosine phosphatase (PTP) that plays an overall positive role in insulin signaling, is linked to the pathogenesis of diabetes and obesity. The relationship between PTP1B and human diseases exhibits PTP1B as the target to treat these diseases. In this article, small weight molecules of the imidazolidine series were screened from databases and optimized on silicon as the inhibitors of PTP1B based on the steric conformation and electronic configuration of thiazolidinedione (TZD) compounds. The top three candidates were tested using an *in vitro* biological assay after synthesis. Finally, we report a novel inhibitor, Compound **13**, that specifically inhibits PTP1B over the closely related phosphatase Src homology 2 (SH2) domain-containing phosphatase 2 (SHP-2) at 80 μM. Its IC_50_ values are reported in this paper as well. This compound was further verified by computer analysis for its ability to combine the catalytic domains of PTP1B and SHP-2 by molecular dynamics (MD) simulations.

## 1. Introduction

Reversible protein tyrosine phosphorylation catalyzed by the coordinated actions of protein tyrosine kinases (PTKs) and protein tyrosine phosphatases (PTPs) is of paramount importance in the regulation of the signaling events that underlie such fundamental processes as growth and proliferation, differentiation and survival or apoptosis, as well as adhesion and motility [1]. PTPs constitute a large, structurally diverse family of receptor-like and cytoplasmic enzymes expressed in all eukaryotes. Scapin G *et al.* [2]. indicated that large numbers of PTP genes were encoded within the human genome, including trans-membrane, receptor-like, and intracellular, non receptor-like enzymes. PTPs have positive (signal-enhancing) or negative (signal-attenuating) roles in a variety of normal signal transductions [3]. And PTPs have been shown to be negative regulators of the insulin receptor. Inhibition of PTPs may be an effective method in the treatment of type 2 diabetes [4].

Protein tyrosine phosphatase 1B (PTP1B), an intercellular non-receptor PTPs, is a key element in the negative regulation of the insulin signaling pathway and a valid potential drug target for the treatment of type 2 diabetes and other associated metabolic syndromes [5,6]. It acts by dephosphorylation of specific phosphotyrosine (pTyr) residues on the insulin receptor and insulin receptor substrate proteins [7]. Zinker *et al.* reported that PTP1B antisense oligonucleotides (ASOs) could reduce PTP1B protein expression and could be used as potential therapeutics in the treatment of type 2 diabetes and obesity [8].

Src homology 2 (SH2) domain-containing phosphatase 2 (SHP-2), another non-receptor PTP, has two Src homology 2 (SH2) domains and a catalytic domain [9,10]. SHP-2 is considered to be a component of several intracellular signal transduction systems involved in embryonic development that modulate cell division, differentiation, and migration, including that mediated by epidermal growth factors [3,10].

The identification of specific small-molecular-weight inhibitors of tyrosine phosphatases is a challenging endeavor, because the base of the catalytic cleft, the signature motif, is highly conserved among all PTPs [11]. Most advanced inhibitors of the tyrosine phosphatase PTP1B, could have some sort of effect on the closely related phosphatase SHP-2 with the same interaction owing to the homology in the targeting sites between PTP1B and SHP-2 [12]. So the inhibitors of PTP1B could, at the same time, affect the activity of SHP-2. Therefore, undoubtedly, a large amount of inhibitors would be needed to acquire the comparable effect by the absence of SHP-2, which might lead to potential toxic and side effects. Troglitazone, a PTP1B inhibitor [13], which is a member of the thiazolidinedione (TZD) compounds, already has been forbidden to be used for the treatment of diabetes in clinical situations in recent years due to its side effects and toxicity [14,15]. Based on the structure and bioavailability of TZD compounds, the database of optimized structures was established on silicon. Therefore, the study of specific PTP1B inhibitors as drugs contributes to the increase of the specific affinity for PTP1B and prevents the combination with protein SHP-2 as far as possible.

**Figure 1:**
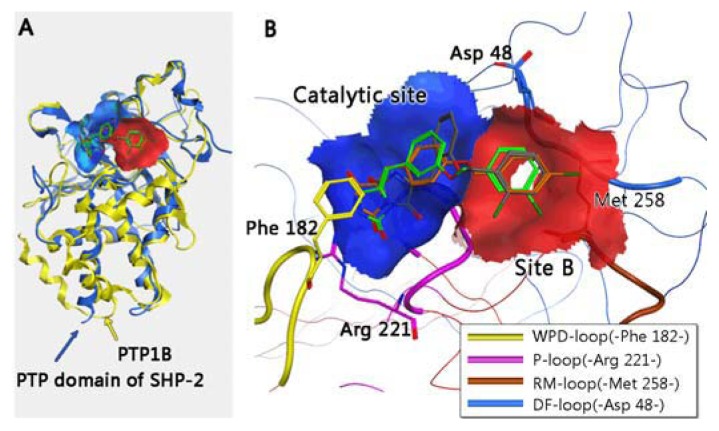
(**A**) The conformational superimposition of proteins encoding the Protein tyrosine phosphatase 1B (PTP1B) and protein tyrosine phosphatase (PTP) domain of SHP-2 with novel ligands and (**B**) the surface map of PTP1B within a 4 Å radius designed around the molecules. The blue surface indicates the catalytic site of the two proteins, while the area colored in red represents site B of PTP1B. Ligands with grey, green and orange colored carbon atoms represent Compounds **13**, **15** and **20**, respectively.

Pei *et al*. [16] reported that the catalytic domain of SHP-2 shared 51% homology with that of PTP1B (as seen in Figure 1). As a result, specific inhibitors against PTP1B are quite difficult to design based on the conformation of the binding pocket of PTP1B. Zhang and co-workers found a second binding active site in 1997, close to the conserved primary active site of PTP1B, also referred to as site B [17]. Furthermore, the residue-type and conformation of site B in PTP1B are not at all the same as those of SHP-2. Thus the aim of achieving selectivity of PTP1B over SHP-2 by taking advantage of the less homologous site B is meaningful and reasonable. Based on the conformation and key residues of the catalytic pocket and site B, we screened the NCI small structures database [18] by Glide5 of the Schrodinger 2009 suit [19] with a larger binding area of PTP1B containing site B to obtain specific inhibitors.

NSC659447, considered as a drug-like structure with a high docking score and good space conformity by NCI database, was optimized by core-hopping [19]. After optimization, we report the synthesis and biological activities of Compounds **13**, **15** and **20**, which have been designed as specific inhibitors of PTP1B. The synthetic scheme and the characterization data are illustrated in the Supplementary data section. The *in vitro* tyrosine phosphatase assay is also shown below. The binding models of Compounds **13**, **15** and **20** with PTP1B and SHP-2 are predicted and analyzed using a molecular dynamics (MD) simulation at the end of this article. The specific inhibitors of PTP1B in this article are not only considered as potential pre-drugs for treating diabetes and obesity but also as probers to discover the effect of PTP1B in the insulin signaling pathway.

## 2. Results and Discussion

### 2.1. Virtual Screening and Core-Hopping

**Figure 2:**
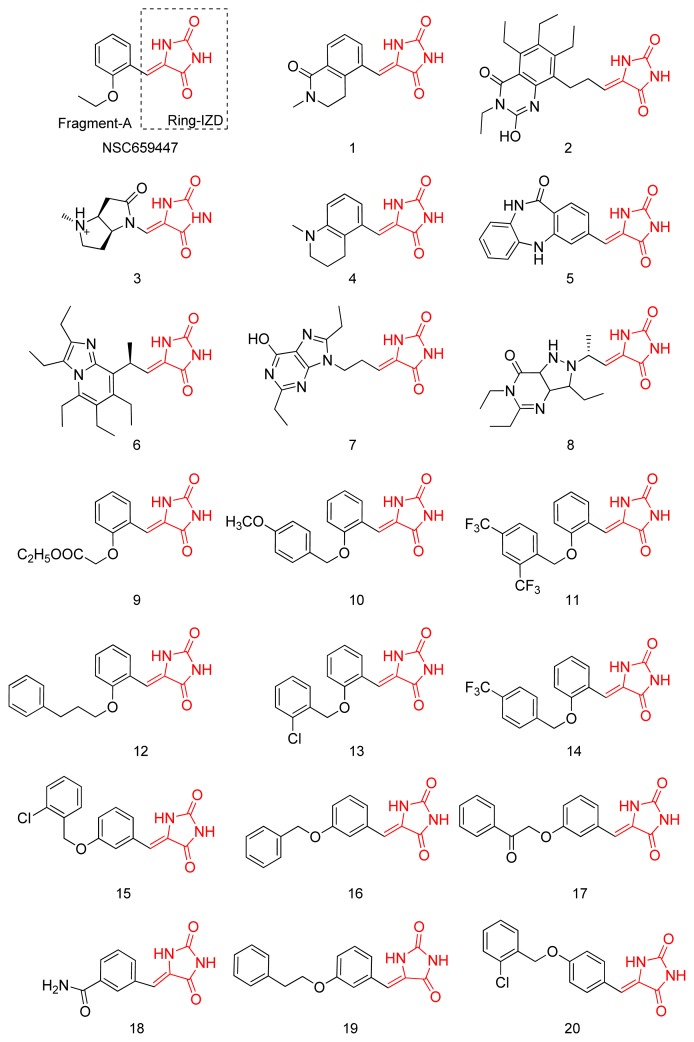
The top 20 derivative compounds offered by method of core-hopping. Ring-IZDs are colored in red; whereas Fragment-A is colored in black, which was replaced by package Core-Hopping.

The database of drug-like structures from NCI [18] was screened by using Glide5 based on the conformation of the catalytic site of PTP1B. NSC659447, found to be the most potential lead compound for further modification, was divided into two parts, Ring-IZD (R-IZD) and Fragment-A (FA) as shown in Figure 2. In order to obtain specific inhibitors of PTP1B over SHP-2, the FA part was replaced by other segments of the fragment database to extend its length to site B. After optimization, the database of 20 candidates was established. Subsequently, each structure of the 20 candidates was redocked into the two receptors, PTP1B and SHP-2, respectively. Figure 2 lists the top 20 derivative candidates.

The candidates are sorted by their binding energies with PTP1B and SHP-2, respectively, in Table 1. The energy was defined as:

(1) $$E_{\text{binding}}=E_{\text{vdw}}+E_{\text{ele}}$$

*E*_vdw_ and *E*_ele_ indicate the binding energy of Van de Waals and electrostatic interaction between the ligand and receptors, respectively. Δ*E* indicates the binding energy of ligand and PTP1B over SHP-2.

The binding energies of the top 20 optimized candidates and NSC659447 with two proteins as well as ΔE are listed in Table 1. Almost all candidates showed higher combined interactions with PTP1B than NSC659447 except Compounds **1** and **18**. Meanwhile, 14 of the 20 candidates exhibited stronger affinities with PTP1B than with SHP-2. ΔE was considered as a key criterion to evaluate the specification of one compound with PTP1B over SHP-2. Compounds **13**, **15** and **20**, as the top three candidates with good ΔE, had the potent specification to inhibit the activity of PTP1B over SHP-2.

### 2.2. Synthesis

The chemical synthesis of the imidazolidine series was achieved by a two-step protocol, as illustrated in Supplementary data. These compounds were synthesized starting from hydroxy substituted benzaldehydes as described previously in Ref. [7,20,21].

### 2.3. Experimental Assay

Compounds **13**, **15** and **20** were tested using the *in vitro* tyrosine phosphatase assay. PTP1B PTP domain GST fusion protein purified in house was used as the enzyme and a phospho-peptide corresponding to the surrounding sequence of pTyr^1146^ in the insulin receptor (Thr-Arg-Asp-Ile-Tyr-[PO_3_H_2_]-Glu-Thr-Asp-Tyr-Tyr) were used as the substrate. This assay determined free phosphate generated by dephosphorylation of the PTP substrate using the Malachite Green reagent. Briefly, 0.15 μg of GST-SHP-2- PTP was incubated in 40 μL assay buffer (25 mM Tris-HCl, PH7.4, 50 mM NaCl, 5 mM DTT, and 2.5 mM EDTA) with test compounds at 80 μM or DMSO in a clear 96 well plate at room temperature for 30 min. The PTP substrate was then added to a final concentration of 0.1 mM. The system was incubated at 30 °C for 30 min. Finally, 50 μL of Malachite Green solution was added and OD_620_ was measured after 15 min [22].

The GST-PTP1B PTP domain fusion protein was incubated with the compounds at room temperature for 30 min before the phospho-peptide substrate was added to the assay systems, allowing compounds to bind to the target site in SHP-2. Each test compound was dissolved in DMSO. The concentration of the test compounds was 80 μM. In the experimental assay, DMSO was included as the negative control.

In the phosphatase assay for SHP-2, procedures were similar to those of PTP1B, with the exception that GST-SHP-2 was used instead as the enzyme.

**Figure 3:**
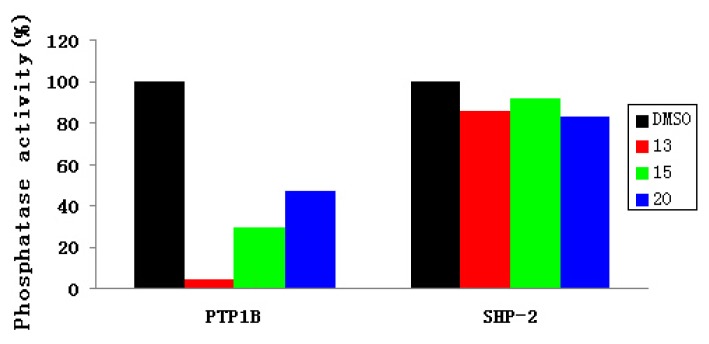
Functional specificity of Compounds **13**, **15** and **20** at the indicated concentrations were subjected to the phosphatase assays using PTP1B and SHP-2 as enzymes. DMSO was used as the negative control.

As shown in Figure 3, at a concentration of 80 μM, Compounds **13**, **15** and **20** inhibited 96%, 70% and 53% of PTP1B enzymatic activity, respectively. However, none of these compounds showed any obvious inhibition of SHP-2. It is worth mentioning that Compound **13** selectively inhibited PTP1B *vs.* SHP-2. Consequently, Compound **13** was considered as a potential inhibitor targeting PTP1B over SHP-2.

The IC_50_ values of the action against PTP1B and SHP-2 of Compounds **13**, **15**, **20** were further determined. The measurements were performed following the experimental protocols:

Human recombinant PTP1B or SHP-2 was expressed in *E. coli* and purified by Ni-NTA affinity chromotagraphy in our laboratory. The enzyme activity was measured using p–nitrophenyl phosphate (pNPP) as substrate in a 96-well plate. Briefly, purified recombinant PTP1B or SHP-2 (0.05 μg) in 50 μL buffer containing 50 mM citrate (pH 6.0), 0.1 M NaCl, 1 mM EDTA, and 1 mM dithiothreitol (DTT) and test compounds were added to each well of a 96-well plate. After pre-incubation for 15 min at room temperature, 50 μL of reaction buffer containing 2 mM pNPP was added and incubated at 37 °C for 30 min. The PTP1B or SHP-2 activity was measured by detecting the absorbance at 405 nm for the amount of produced *p*-nitrophenol.

The IC_50_ values against PTP1B and SHP-2 are reported in Table 2. Impressively, Compound **13** shows moderate selectivity to PTP1B over SHP-2, and the results obtained are consistent with the tested percentage inhibition above.

## 3. Molecular Dynamics Simulations

Molecular dynamics (MD) simulations on silicon were used to obtain functional information in order to characterize the interactions between ligands (Compounds **13**, **15** and **20**) and proteins (PTP1B and SHP-2). In this case, a number of 10 ns MD simulations was performed to estimate the differences of binding affinity and interaction between Compound 13 and proteins (PTP1B and SHP-2).

All MD simulations in this article were performed with GROMACS 96-53a6 force fields [23] with the periodic boundary conditions (PBC) using the GROMACS 4.0 package. The topology files and charges for the ligand atoms were generated by the Dundee PRODRG3.0 server [24]. The models in simulations were solvated by explicit simple point charge (SPC) water of 1.0 nm thickness in a cubic box. The system was neutralized with charged ions to replace the SPC water molecules. Subsequently, an energy minimization was performed for the system concerned using the steepest descent until reaching a tolerance of 100 kJ/mol. After that, the 10 ns MD simulations were carried out with a time step of 1 fs; the corresponding coordinates were stored every 100 fs. The PME algorithm was used to calculate the electrostatic interactions. All simulations were run under the periodic boundary condition with the NVT ensemble using Berensen’s coupling algorithm to keep the temperature at 310 K and the pressure at 1 atm. All bonds were constrained by using the LINCS algorithm. The GROMACS 4.0 package was utilized to analyze the result.

### 3.1. Molecular Dynamics Trajectory Analysis

**Figure 4:**
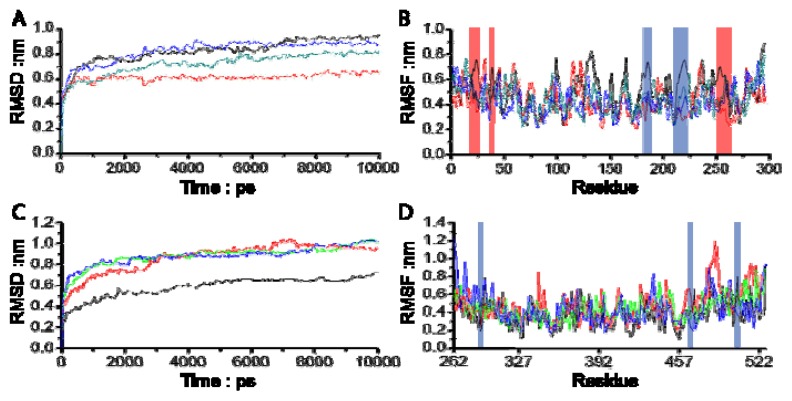
Analysis of the molecular dynamics simulations. (**A**) The The root mean square deviation (RMSD) for all backbone atoms in four simulations of PTP1B system; (**B**) The root mean square fluctuations (RMSF) for side-chain atoms in four simulations of the PTP1B system; (**C**) The RMSD for all backbone atoms in four simulations of the SHP-2 system; (**D**) The RMSF for side-chain atoms in four simulations of the SHP-2 system. The curves colored in grey indicate the systems of PTP1B (**A**,**B**) and SHP-2 (**C**,**D**) without any compounds, while the curves of PTP1B (**A**,**B**) and SHP-2 (**C**,**D**) with the novel Compounds **13**, **15** and **20** are colored in magenta, green and blue, respectively. The curves associated with catalytic site and site B in Figure 4B,D are indicated by blue and red squares, respectively.

The root mean square deviation (RMSD) from the initial conformation is a central criterion used to evaluate the transformation of the protein system. The stability of a simulation system was evaluated based on its RMSD as well. The RMSD values for all four systems *versus* the simulation time are illustrated in Figure 4A, in which all four systems meet the stable state after 3 ns. In the stable state, all complex systems of PTP1B with Compounds **13**, **15** and **20** were significantly more stable than PTP1B uncomplexed with the compound, and more remarkably, the RMSD value for PTP1B complexed with Compound **13** was much smaller than that of the other three systems, indicating that the flexibility of PTP1B is restricted when complexed with Compound **13**. As can be seen in Figure 4C, SHP-2 complexed with all three compounds reached the stable state after 3 ns too, however, the RMSD of three complex systems reached the same level, which was around 0.9 nm, while the RMSD of the related protein uncomplexed with the compound was 0.6 nm during the stable state. It was revealed that Compounds **13**, **15** and **20** did not restrict the transformation of the catalytic site of SHP-2. These results are consistent with the observation in the biological experiment.

In order to investigate the motions of the important residues interacting with the inhibitors in the binding sites defined as catalytic site and site B in Figure 1, the root mean square fluctuations (RMSF) for all the side chain atoms of the two proteins were calculated, as shown in Figure 4B,D. It can be clearly seen from Figure 4B that the fluctuating magnitudes of PTP1B uncomplexed with the compound (grey curve) in the catalytic site (blue square) and site B (red square) are much larger than those in the complex systems with three different compounds. The fluctuating magnitudes of residues **Cys215**-**Arg221** in the catalytic site reached the lowest point when binding Compound **13**, which was around 0.3 nm. Those in the complex systems with Compounds **15** and **20** were quite similar, approximately 0.4nm, while the RMSF of loop **Arg254-Met258** in site B of PTP1B uncomplexed with compound was nearly 0.7 nm, and therefore much larger than that in the complex systems. At the same time the fluctuating magnitudes of key residues **Arg24** and **Ile219** in the complexes’ trajectories were much smaller than that in PTP1B trajectory as well.

As expected, all three inhibitors could steadily occupy both the catalytic site and site B with loop **Arg254-Met258** as well as the key residues **Arg24** and **Ile219** of PTP1B, and effectively restrict the motions of the related key residues. However no significant change in simulations of protein SHP-2 with inhibitors were found in Figure 4D. Compounds **13**, **15** and **20** had no capacity to restrict the key residues around the catalytic site, revealing that they could not inhibit the activity of SHP-2. Of all three compounds, Compound **13** was anticipated to be a promising drug candidate of PTP1B, exactly the same as in the biological assay.

### 3.2. Combinational Ways and Energy Analysis during the Simulations

In order to evaluate the stability of the combination in this article, RMSF for all the heavy atoms of small molecules were recorded. As can be seen in Table 3, the fluctuation of Compound **13** in the pocket of PTP1B was the lowest, with a RMSF of 0.07 nm. This phenomenon revealed that Compound **13** could steadily occupy the active pocket of PTP1B. On the other hand, the performance of Compound **13** in the binding site of SHP-2 was not good, with a RMSF of 0.64 nm that far outweighed that in PTP1B. In addition, the RMSF of Compounds **15** and **20** were 0.49 and 0.33 nm in PTP1B, and 0.85 and 0.67 nm when binding to the active site of SHP-2, respectively, indicating that all three compounds were able to steadily occupy the pocket of PTP1B over SHP-2 against the phosphorylated substrate’s combined approach to the active site.

Average distances between the chlorobenzyl group of the ligands and the central point of site B (DLB) in protein PTP1B were calculated to evaluate the stability of the end group of the small molecule, chlorobenzyl group, in site B of PTP1B. The central point of site B was defined by four residues (**Arg24**, **Asp48**, **Ile219** and **Met258**) with low RMSF during the simulations located at higher points surrounding the valley of site B, while the central point of the chlorobenzyl group in the molecule was the central point of all involved heavy atoms in this group. The stability of the chlorobenzyl group binding site B was performed by using distance mean value and variance. As seen in Table 2, the chlorobenzyl group of Compound **13** combined with site B of PTP1B was much steadier than that in any other system, with a DLB of 0.70 ± 0.11 nm, indicating that Compound **13** was able to steadily occupy the pocket of PTP1B during the simulation to inhibit the activity of PTP1B.

We divided the binding energy into two parts, contributions of the catalytic site and site B. Binding energy was generated by the interactions of Van der Waals forces and Coulomb forces between one compound and certain parts of the protein. The results were calculated using the energy mean value and variance during the molecular dynamics simulations. As illustrated in Table 3, the binding energy for the catalytic site of PTP1B basically approached that calculated for SHP-2, on the other hand, the binding energy with site B in protein PTP1B was quite different from that in protein SHP-2. This phenomenon indicated that the main specific binding interaction with PTP1B might be determined by interactions between the ligand and site B. These results are consistent with the biological assay.

### 3.3. Residues Involved in the Interaction between Ligand and Key Residues

To obtain an improved understanding of the energy profile, the binding energy was decomposed into groups of key residues in different site interactions. It is clear from Tables 4 and 5 that the P-loop (**Cys215**-**Arg221** in PTP1B and Cys220-Arg226 in SHP-2, numeration referred to SHP-2 is in italics in order to distinguish it from the numeration referring to PTP1B) provided the highest contribution to the binding, indicating that these compounds tightly tied the key residues located in the catalytic site during the simulations and inhibited the catalytic activity as a result of satiric hindrance. On the other hand, in the area of site B of PTP1B, the DF-loop (**Asp48-Phe52**) contributed a larger binding energy than the NF-loop (Asn20-Phe24) in SHP-2, suggesting that the DF-loop was indeed involved in binding compounds and especially contributed to the selectivity of PTP1B for Compound **13**.

## 4. Material and Methods

### 4.1. Starting Structures

**Figure 5:**
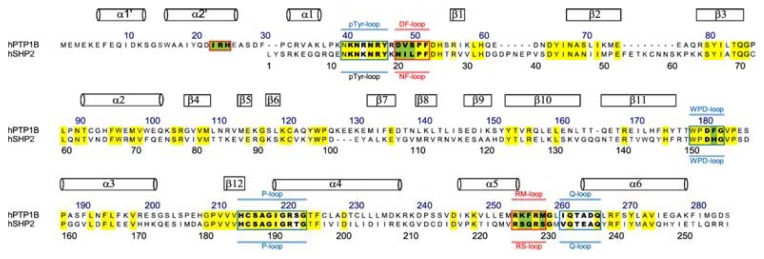
Sequence alignment of PTP domains between PTP1B and Src homology 2 (SH2) domain-containing phosphatase 2 (SHP-2). The catalytic site and site B regions are boxed with blue and red lines, respectively. In this article, single letters with yellow and green squares indicate the conserved residues and non-conserved residues involved in the definition of substrate selectivity-determining regions. Residue numbers for PTP1B and SHP-2 are shown above and below the alignment, respectively.

The X-ray crystal structure of PTP1B with the inhibitor 1NTB at a 2.15 Å resolution was downloaded from the RSCB website [25], (PDB file: 2QBQ [26]). The combined area between PTP1B and 1NTB in the crystal structure of 2QBQ was considered as the binding site for screening the database of small structures. The catalytic pocket of PTP1B was made up of three main segments (P-loop, WPD-loop and Q-loop) [27]. Zhang and co-workers first identified a second phosphotyrosine (pTyr) binding site (site B) in the vicinity of the PTP1B active site [17] as seen in Figure 1B and Figure 5, which contains several hydrophobic and hydrophilic residues for potential binding interactions (**Asp48**, **Val49**, **Phe52**, **Ile219**, **Met258**, **Arg24** and **Arg254**) (Numeraction referred to PTP1B is in bold). A grid box of PTP1B for virtual screening was formed by residues around the catalytic pocket and site B.

SHP-2 is a protein tyrosine phosphatase (PTPs). According to previous reports [28], the catalytic activity of SHP-2 is auto-inhibited by its *N*-SH2 and *C*-SH2 domains. Therefore, the crystal structure of SHP-2 with an accessible active site was obtained from the RSCB PDB bank, (PDB ID 3B7O) [29]. This enzyme comprises the entire catalytic PTP and *C*-tail domains without the *N*-SH2 and *C*-SH2 domains [30]. The binding pocket was formed by those residues that had at least one heavy atom (*i.e.*, an atom other than hydrogen) with a distance of <5 Å from any heavy atom of its original ligand D-MALATE.

The small structures database and fragments database derived from NCI [18] and ZINC [31] were also employed to research the virtual screening and optimization.

### 4.2. Virtual Screening and Core-Hopping Procedure on Silicon

The database of small structures from NCI [18] was screened by the Glide5 docking package of the Schrodinger suite 2009 [19] based on the 3D structure of residues in the grid box of PTP1B. Protein PTP1B and small structures were geared up by Protein Preparation Wizard and LigPrep modules embedded in Schrodinger suite 2009 (www.schrodinger.com [32]), respectively. For protein preparation, the process included assigning bond orders, adding hydrogen, treating metals, treating disulfides, deleting water and alleviating potential steric clashes, adjusting bond order and formal charges by protein minimization with the OPLS2005 force field [33]. The constrained refinement value of root mean square deviation (RMSD) for the protein was limited to 0.3 Å. Meanwhile, for the compounds, the preparation consisted of generating possible states by ionization at a target pH of 7.0, desalting, retaining chiralities from 3D structure and geometry minimization with the OPLS2005 force field as well [33]. When the above steps were accomplished, all investigated structures in the NCI database were docked into the receptor pocket through the rigid docking model with the stand-precision (SP) scoring function [34,35] to estimate the binding affinities.

In order to gain high binding affinity inhibitors of PTP1B over SHP-2, the program for Core-Hopping [36,37] in Schrodinger suite 2009 was utilized in this study that has the function to perform both the fragment-based replacing and molecular docking. During the process of core-hopping, the point was defined in advance for scaffold replacement in the Define Combinations Step from the Combinatorial Screening panel. After setting the receptor grid in the Receptor Preparation panel, the novel structures were obtained by scaffold-replacement and then re-docked into the receptors.

## 5. Conclusions

We have reported on the PTP1B selective inhibitors using CADD combined with experimental assays. Compound **13** of the 20 computationally selected compounds showed inhibitory effects on PTP1B catalytic activity in the *in vitro* phosphatase assay. It also selectively inhibited PTP1B over SHP-2. Our study provides a novel scaffold upon which more potent and selective PTP1B inhibitors could be developed through structural modifications, extending to site B for more interactions with the DF-loop. Further research studying the inhibition mode following this molecule design will be described in the future. The availability of PTP1B selective inhibitors could offer a decent contribution to the development of novel structures of drug to treat PTP1B associated diseases, it may, however, also facilitate research on PTP1B involved insulin signaling in model systems.

The current structure-based drug discovery approach, involving multiple computational techniques, should also be applicable to identify the selectivity of small molecule inhibitors of other PTPs.

## Supplementary Information



## Figures and Tables

**Table 1:** Binding energies of 20 derivative compounds with PTP1B and SHP-2 (kcal/mol).

Compound	*E*_PTP1B_	*E*_SHP-2_	Δ*E*
**13**	−46.76	−34.41	12.35
**20**	−42.37	−32.98	9.38
**6**	−41.03	−32.30	8.73
**15**	−40.83	−29.03	11.80
**14**	−40.75	−37.02	3.73
**7**	−40.75	−36.33	4.42
**16**	−40.36	−31.62	8.74
**19**	−40.24	−33.79	6.44
**5**	−39.99	−40.23	-
**17**	−39.50	−32.89	6.61
**2**	−38.57	−39.03	-
**11**	−38.35	−34.17	4.18
**8**	−37.89	−31.22	6.67
**12**	−35.78	−27.98	7.80
**10**	−36.79	−38.20	-
**3**	−36.43	−29.90	6.52
**9**	−35.57	−36.96	-
**4**	−33.76	−34.78	-
**1**	−33.03	−34.02	-
**18**	−32.78	−28.22	4.56
NSC659447	−33.34	−25.19	8.15

**Table 2:** Selective inhibitory activity against PTP1B and SHP-2.

IC_50_ (μM)	13	15	20
PTP1B	37.4	57.3	63.9
SHP-2	152	152	>152

**Table 3:** Combinational mode and energy analysis during the simulations (kcal/mol).

	PTP1B				Catalytic site of SHP-2		
		
	RMSF ^c^ (nm)	DLB ^d^ (nm)	Catalytic site (Kcal/mol)	Site B (Kcal/mol)	RMSF ^c^ (nm)	Catalytic Site (kcal/mol)	Site B (kcal/mol)
**13**	0.07	0.70 ± 0.11	−39.30 ± 2.80	−34.30 ± 2.14	0.64	−43.73 ± 5.18	−8.3 ± 2.17
**15**	0.49	1.10 ± 0.28	−42.00 ± 6.39	−20.74 ± 4.51	0.85	−45.97 ± 6.39	−3.5 ± 1.76
**20**	0.33	0.86 ± 0.30	40.70 ± 7.92	−24.67 ± 5.12	0.67	−42.27 ± 4.92	−4.9 ± 2.66

RMSF ^c^: the root mean square fluctuations for heavy atoms of small molecule during the simulations; DLB ^d^: average Distances between Ligands and central point of site B in protein PTP1B during the simulations.

**Table 4:** The key residues of the PTP1B binding energy during the simulation (kcal/mol).

PTP1B	Catalytic Site			Site B		
	
	-Phe182-	P-loop ^e^	Q-loop ^f^	-Arg 24-	DF-loop ^g^	RM-loop ^h^
**13**	−4.25	−37.84	−4.65	−5.84	−13.23	−2.35
**15**	−5.13	−29.88	−3.53	−4.66	−9.10	−2.32
**20**	−4.49	−30.36	−5.28	−1.50	−9.29	−3.50

P-loop ^e^: Cys215-Arg221; Q-loop ^f^: Gln262-Gln266; DF-loop ^g^: Asp48-Phe52; RM-loop ^h^: Arg254-Met258.

**Table 5:** The key residues of the SHP-2 binding energy during the simulation (kcal/mol).

SHP-2	Catalytic Site			Site B	
	
	-His154-	P-loop ^i^	Q-loop ^j^	NF-loop ^k^	RS-loop ^l^
**13**	−3.50	−25.32	−3.35	−3.18	−1.71
**15**	−1.27	−19.62	−7.01	−1.25	−0.11
**20**	−0.78	−23.84	−6.98	−1.07	−0.28

P-loop ^i^: Cys220-Arg226; Q-loop ^j^: Gln234-Gln238; NF-loop ^k^: Asn20-Phe24; RS-loop ^l^: Arg226-Ser230.
