# Supplementary material for: Design, Synthesis, Biological Activity and Molecular Dynamics Studies of Specific Protein Tyrosine Phosphatase 1B Inhibitors over SHP-2
Source: Int J Mol Sci. 2013 Jun 17;14(6):12661–74. doi: 10.3390/ijms140612661 (PMC3709806; doi:10.3390/ijms140612661)
Supplement: Supplementary file 1 [file ijms-14-12661-s001.pdf]

Supplementary Information

The synthetic pathways of the top three compounds are illustrated briefly in Scheme 1. Compounds **1a-1c** were obtained in high yields through the nucleophilic substitution reaction of hydroxy substituted benzaldehydes with 1-chloro-(2-chloromethyl) benzene in *N*,*N*-dimethyl formamide (DMF) in the presence of potassium carbonate. After letting the mixture react for 1h and crystallization from dichloromethane/methanol, the desired Compounds **1a-1c** were obtained as single products.

Scheme 1. Synthesis of Compounds **13**, **15** and **20**. Reagents and conditions: (I) 1-chloro-(2-chloromethyl)benzene, K_2CO_3, DMF, 50–85 °C 1 h; (II) 2,4-imidazolidine, piperidine, HOAc, toluene, reflux, 3 h.

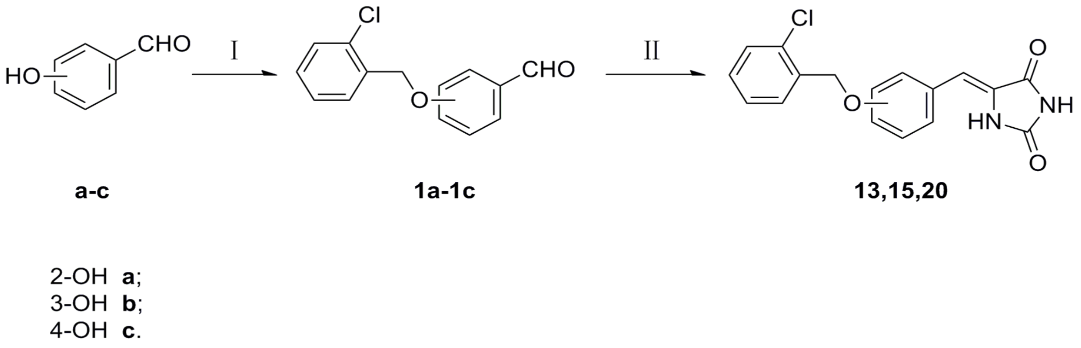

Compounds **13**, **15** and **20** were obtained by the condensation of commercially available 2,4-imidazolidine with **1a-1c** in refluxing toluene in the presence of piperidine and acetic acid, as already reported, but with slightly lower yields. The appropriate piperidine and acetic acid were added in a drop-wise fashion at room temperature and the resulting mixture was refluxed for 3 h. The crude products were recrystallized from dichloromethane/methanol providing pure **13**, **15** and **20**.

1. Chemistry

Reaction courses were monitored by TLC on silica gel pre-coated F254 Merck plates. Developed plates were examined with UV lamps (254 nm). Melting points were measured with an X-6 melting point apparatus. ^{1}H-NMR and ^{13}C-NMR spectra were taken on a Bruker spectrometer using TMS as the internal standard. Mass spectra were measured on an Agilent 1100 series. DMSO-d_6 and $CDCl_3$ were used as the solvents. The major chemicals were purchased from Nanjing TianZunZeZhong Chemical Ltd Co. (Nanjing, China) and Shanghai JingChun Reagents Ltd Co. (Shanghai, China).

Unless stated otherwise, all materials were obtained from commercial suppliers and used without further purification.

2. General Procedure for the Synthesis of 1a-1c

Potassium carbonate (2.07 g, 15 mmol) and substituted benzyl chlorides (20 mmol) were added in a drop-wise manner to a solution of **a**, **b** or **c** (1.83 g, 15 mmol) in 50 mL of DMF at 50 °C. After the addition the solution was allowed to warm to 85 °C. The reaction mixture was stirred for 1 h, diluted with EtOAc, washed with water and saturated brine, dried over anhydrous $MgSO_4$, and filtered. The solvent was then removed via rotary evaporation. The resulting residue was purified by silica gel

column chromatography (dichloromethane/methanol, 20:1 *v*/*v*). The yield, melting point and spectral data of each compound are given below.

*2.1. 2-((2-chlorobenzyl)oxy)benzaldehyde (**1a**)*

Yield 89%; m.p.77.7–78.5 °C. ^{1}H-NMR ($CDCl_3$, 400 Hz), *δ*: 5.299 (s, 2H, OCH_2); 7.078 (t, *J* = 8.0 Hz, 2H, Ar-H); 7.315(q, *J* = 7.2 Hz, 2H, Ar-H); 7.429 (t, *J* = 9.2 Hz, 1H, Ar-H); 7.551 (q, *J* = 6.8 Hz, 2H, Ar-H); 7.886 (t, *J* = 6.8 Hz, 1H, Ar-H); 10.589 (s, 1H, CHO). ^{13}C-NMR ($CDCl_3$, 75 Hz), *δ*: 189.608, 160.721, 135.950, 133.800, 132.685, 129.571, 129.403, 128.741, 128.697, 127.129, 125.271, 121.289, 113.040, 67.727. MS m/z: 247(M + 1), 264(M + NH_4).

*2.2. 3-((2-chlorobenzyl)oxy)benzaldehyde (**1b**)*

Yield 93%; m.p.40.0–41.0 °C. ^{1}H-NMR ($CDCl_3$, 400 Hz), *δ*: 5.227 (s, 2H, OCH_2); 7.259–7.311 (m, 3H, Ar-H); 7.411–7.569 (m, 5H, Ar-H); 9.990 (s, 1H, CHO). ^{13}C-NMR ($CDCl_3$, 75 Hz), *δ*: 192.026, 159.077, 137.876, 134.048, 132.802, 130.214, 129.520, 129.281, 128.874, 127.031, 123.820, 121.994, 113.512, 67.384. MS m/z: 264(M + NH_4).

*2.3. 4-((2-chlorobenzyl)oxy)benzaldehyde (**1c**)*

Yield 89%; m.p.49.9–50.7 °C. ^{1}H-NMR ($CDCl_3$, 400 Hz), *δ*: 5.243 (s, 2H, OCH_2); 7.082–7.876 (m, 8H, Ar-H); 9.899(s, 1H, CHO). ^{13}C-NMR ($CDCl_3$, 75 Hz), *δ*: 190.743, 163.408, 133.687, 132.721, 132.033(d), 130.352, 129.554, 129.397, 128.799, 127.093, 115.136(d), 67.361. MS m/z: 247(M + 1).

3. General Procedure for the Synthesis of 13, 15 and 20

A mixture of **1a**, **1b** or **1c** (2.47 g, 10 mmol), appropriate 2,4-imidazolidine (1.20 g, 12 mmol) in 40 mL of toluene was treated with 4 mL piperidine and 3 mL acetic acid. The reaction mixture was heated at reflux for 3 h and then concentrated via rotary evaporator to afford the crude product. The residue was dissolved in ethyl acetate, washed with 5% hydrochloric acid, saturated sodium bicarbonate and saturated brine, dried over anhydrous $MgSO_4$. And the solvent was removed under reduced pressure. The resulting residue was purified by silica gel column chromatography (dichloromethane/methanol, 20:1 *v*/*v*) to obtain the pure solid sample.

*3.1. (Z)-5-(2-((2-chlorobenzyl)oxy)benzylidene)imidazolidine-2,4-dione (**13**)*

Yield 45%; m.p.199.9–200.8 °C. ^{1}H-NMR(DMSO-d_6, 400 Hz), *δ*: 5.246 (s, 2H, OCH_2); 6.680 (s, 1H, =CH); 7.020 (d, *J* = 8.8 Hz, 2H, Ar-H); 7.166 (d, *J* = 8.4 Hz, 2H, Ar-H); 7.341–7.425 (m, 3H, Ar-H); 7.535–7.636 (m, 3H, Ar-H); 10.429(s, 1H, NH); 11.207(s, 1H, NH). ^{13}C-NMR ($CDCl_3$, 75 Hz), *δ*: 165.900, 156.395, 155.932, 134.524, 133.189, 130.593, 130.492, 130.417, 129.975, 129.838, 128.673, 127.879, 122.424, 121.563, 112.952, 102.998, 67.859. MS m/z: 329(M+1), 327(M − 1), 657(2M + 1).

*3.2. (Z)-5-(3-((2-chlorobenzyl)oxy)benzylidene)imidazolidine-2,4-dione (**15**)*

Yield 30%; m.p.196.5–197.6 °C. ^{1}H-NMR(DMSO-d_6, 400 Hz), δ: 5.234 (s, 2H, OCH_2); 6.403 (s, 1H, =CH); 7.001 (d, J = 8.0 Hz, 1H, Ar-H); 7.221–7.419 (m, 5H, Ar-H); 7.527 (d, J = 4.8 Hz, 1H, Ar-H); 7.630 (d, J = 4.0 Hz, 1H, Ar-H); 10.622 (s, 1H, NH); 11.264 (s, 1H, NH). ^{13}C-NMR ($CDCl_3$, 75Hz), δ: 165.974, 158.902, 156.185, 134.841, 134.766, 133.129, 130.704, 130.358, 130.337, 129.861, 128.606, 127.841, 122.929, 115.620, 115.443, 108.571, 67.378. MS m/z: 329(M + 1), 327 (M − 1), 346(M + NH_4).

*3.3. (Z)-5-(4-((2-chlorobenzyl)oxy)benzylidene)imidazolidine-2,4-dione (**20**)*

Yield 30%; m.p.257.1–258.2 °C. ^{1}H-NMR(DMSO-d_6, 400 Hz), δ: 5.216 (s, 2H, OCH_2); 6.400 (s, 1H, =CH); 7.063 (d, J = 8.8 Hz, 2H, Ar-H); 7.414 (q, J = 7.6 Hz, 2H, Ar-H);7.524–7.547 (m, 1H, Ar-H); 7.612(d, J = 8.8 Hz, 3H, Ar-H); 10.453(s, 1H, NH); 11.184(s, 1H, NH). ^{13}C-NMR ($CDCl_3$, 75 Hz), δ: 166.045, 158.764, 156.086, 134.523, 133.166, 131.579(d), 130.732, 130.447, 129.888, 127.857, 126.744, 126.401, 115.514(d), 108.927, 67.433. MS m/z: 329(M + 1), 327(M − 1).

© 2013 by the authors; licensee MDPI, Basel, Switzerland. This article is an open access article distributed under the terms and conditions of the Creative Commons Attribution license (http://creativecommons.org/licenses/by/3.0/).
